# Manual of the Diseases of Children

**Published:** 1901-05

**Authors:** 


Manual of the Diseases of Children. By John Madison Taylor, A. M., M. D., Professor of Diseases of Children, Philadelphia Polyclinic; Assistant Physician to the Children's Hospital and to the Orthopedic Hospital; Neurologist to the Howard Hospital, etc. And William H. Wells, M. D., Adjunct Professor of Obstetrics and Diseases of Infancy, Philadelphia Polyclinic; Instructor in Obstetrics in the Jefferson Medical College. Octavo, pp. 859. Illustrated. Second edition, thoroughly revised and enlarged. Philadelphia: P. Blakiston's Son & Co. 1901. [Price, $4.50.]

The second edition of this work is much larger than the first, some chapters having been added outright, the entire volume revised and many minor additions made. Some features deserve special mention. A short chapter on symptomatology and diagnosis is excellent. The chapter on infant feeding has been thoroughly revised. The directions for the home modification of milk give the chapter great practical value, being clear and concise and easy to follow even in a humble home.

Among the new subjects taken up in this edition is a simple practical consideration of diseases of the ear. Every physician who reads it will be glad of the addition. The chapter on the blood has been entirely rewritten and is practically new. The chapter on physical development is a most readable one; especially is that section of it devoted to feebleness in girls at the age of puberty replete with wise counsel.

The American writers on pediatrics have raised a high standard, and this present edition ranks among the best. M. J. F.

